# Screening and Characterization of the Diversity of Food Microorganisms and Their Metabolites

**DOI:** 10.3390/microorganisms11051235

**Published:** 2023-05-08

**Authors:** João Miguel Rocha, Biljana Kovacevik, Sanja Kostadinović Veličkovska, Mercedes Tamame, José António Teixeira

**Affiliations:** 1 CBQF-Centro de Biotecnologia e Química Fina—Laboratório Associado, Escola Superior de Biotecnologia, Universidade Católica Portuguesa, Rua Diogo Botelho 1327, 4169-005 Porto, Portugal; 2 LEPABE—Laboratory for Process Engineering, Environment, Biotechnology and Energy, Faculty of Engineering, University of Porto, Rua Dr. Roberto Frias, s/n, 4200-465 Porto, Portugal; 3 ALiCE—Associate Laboratory in Chemical Engineering, Faculty of Engineering, University of Porto, Rua Dr. Roberto Frias, s/n, 4200-465 Porto, Portugal; 4 Faculty of Agriculture, University “Goce Delčev”, Krste Misirkov bb, 2000 Štip, North Macedonia; biljana.kovacevik@ugd.edu.mk (B.K.); sanja.kostadinovik@ugd.edu.mk (S.K.V.); 5 Institute of Functional Biology and Genomics (IBFG), CSIC-University of Salamanca, 37007 Salamanca, Spain; tamame@usal.es; 6 Centre of Biological Engineering, University of Minho, 4710-057 Braga, Portugal; jateixeira@deb.uminho.pt

**Keywords:** LAB, fermentation, antimicrobial properties, bacteriocins, antioxidants

## Abstract

Food is rarely kept in a sterile environment and the composition of microbial associations found in various foodstuffs is widely varied. Microorganisms in food usually originate from the natural microbiota of raw materials and the surrounding environments. Whether a species prevails depends upon its ability to adapt to intrinsic factors associated with foods, such as nutrient content; pH; water activity; oxidation–reduction potential; and antimicrobial properties, with various extrinsic factors playing a role, including temperature, relative humidity, atmosphere, and ambient pressure. Any change to these parameters may cause changes in the present microbial consortia. Therefore, it is important to identify which microbial consortia will thrive in particular foods and conditions. While active, microorganisms undergo many complex mechanisms that affect food quality and safety. Most beneficial food microorganisms belong to lactic acid bacteria and yeasts. Pathogenic and spoilage bacteria are usually Gram-negative, although there are some Gram-positive ones, such as *Listeria monocytogenes*, *Clostridium botulinum,* and *C. perfringens*. Some may merely cause spoilage, while others may be related to foodborne illnesses.

## 1. Introduction

Food microorganisms can produce valuable compounds, such as organic acids, exopolysaccharides (EPS), peptides (especially bacteriocins), bacteriocin-like inhibitory substances (BLIS), short-chain fatty acids (SCFA), vitamins, ethanol, aroma compounds, some other low-molecular-weight compounds, etc. [1]. The type of metabolites and their relative ratios depend on the synergistic action between the genomes of microorganisms and the environmental conditions in which they grow [2]. Some compounds possess antimicrobial and antioxidative properties, thus enhancing the safety and stability of foodstuffs, and have also been proven to be suitable for use in the food industry. Lactic acid bacteria naturally dominate the microbiota of many foods and have a major potential for use in food biopreservation; their consumption is safe since they do not have lipopolysaccharides in their cell membrane; thus, anaphylactic shock is avoided when administered in humans [3]. Their use is especially beneficial in fermented food, where they usually are found in consortia with yeasts and acetic acid bacteria (AAB). They can be naturally present or introduced as pure cultures. Their metabolic activities contribute to the natural biopreservation of such foods and the extension of shelf-life [4]. The antimicrobial and antioxidant properties of LABs are particularly important for food engineering since prolonged exposure to chemical preservatives may pose health risks in humans, so the use of such natural antimicrobial preservatives is highly desirable to consumers and are likely to be used in clean-label foodstuffs. The antimicrobial properties of LAB may appear indirectly by lowering the pH, changing the osmotic pressure in the food, or directly producing numerous antimicrobial compounds via complex metabolic pathways. In fact, these processes are not independent of one another; thus, the decrease in pH usually occurs because of microbial metabolisms and the production of mostly organic acids and ethanol.

## 2. Special Issue on “Screening and Characterization of the Diversity of Food Microorganisms and Their Metabolites”

Beneficial microorganisms, such as lactic acid bacteria (LAB) and yeasts (especially those from the genus *Saccharomyces),* are highly appreciated in the food and beverage industry alongside home-prepared foodstuffs since they contribute to the processes of fermenting sugars into other valuable substances. The two-stage fermentation of cider and wine—which starts with the alcoholic fermentation of sugars by yeast from the *Saccharomyces* species and then continues with acetification where ethanol is oxidized to acetic acid, generally involving species from the family of acetic acid bacteria—contributes to vinegar production [5]. Various compounds from vinegar, generated during the Millard reaction, are identified to have antioxidant properties [6]. Therefore, the antioxidant capacity of vinegar will depend on the type of fruit employed to prepare it [7].

The role of LAB and yeasts in bread preparation is invaluable, especially when it is traditionally prepared, such as with sourdough. The spontaneous fermentation of flour and water in the sourdough formation establishes highly diverse microbial communities that are beneficial for human health due to naturally present microorganisms in flour and the surrounding environment. Considering that each sourdough is unique, Lau et al. [8] give an overview of microbial communities isolated from sourdough prepared from different kinds of flours. The investigation showed that most of the microorganisms isolated from sourdough prepared from flour originating from different types of cereals belong to the genus *Lactobacillus*, with *Lactiplantibacillus plantarum* found to be the most abundant followed by *Levilactibacillus breavis and Lacticaseibacillus casei.* Other less present bacterial species found belonged to the genera *Weissella*, *Enterococcus*, *Pediococcus,* and *Aerococcus.* The most abundant yeast was *Saccharomyces cerevisiae,* followed by *Candida humulis*. Still, other present yeast species belonged to the genera of *Kazachstania*, *Pichia*, *Wickerhamomyces*, *Rhodotorula,* and *Torulaspora*. There are numerous benefits of these microorganisms for the properties of sourdough bread. They contribute to better rheological and sensory characteristics of bread and extended shelf life due to their metabolites, such as various organic acids, which also possess antimicrobial properties against the most deleterious food spoilage microorganisms, such as *Penicillium*, *Mucor*, *Aspergillus*, *Fusarium*, *Monillia*, etc. [8]. Understanding the fermentation patterns of LABs and yeasts allows us to select strains that can be used as commercial sourdough starter cultures with the desired and well-tailored properties. Petkova et al. [9] investigated LAB and yeast species from typical Bulgarian sourdoughs originating from various geographical locations in Bulgaria to select strains that can be used as commercial sourdough starter cultures. Based on their acid production capacity, amylolytic and proteolytic properties, and antimicrobial properties, strains such as *Lb. brevis* 01M22, *Lb. brevis* 07B198, *Lb. plantarum* 08B217, and *P. pentosaceus* 12R2192 were found to be the most promising for the development of new active starter cultures with good biopreservation capacities. Müller et al. [10] investigated 99 strains from the genus *Leuconostoc* isolated from 2 sourdough starters for their antimicrobial activity and potential to be used as non-conventional starter cultures in sourdough fermentation. For the investigation, four *Lc. Citreum* strains (DCM49, DCM65, MA079, and MA113) were selected for lab-scale sourdough fermentation. The results revealed that *Lc. citreum* possesses a high potential for being used as multiple techno-functional starter cultures in sourdough fermentations. In addition to the benefits related to the rheological quality and sensory characteristics of bread, these microorganisms give many benefits to human health, especially those related to the gastrointestinal system. The proteolytic activity of LAB and their potential to contribute to better bread digestibility and other bakery products are beneficial for patients suffering from non-celiac wheat sensitivity or irritable bowel syndrome. Fraberger et al. [11] investigated the degradation potential of eighty-seven LAB isolates obtained from traditional Austrian sourdoughs, the Belgian Coordination Collection of Microorganisms (LMG), and the German Collection of Microorganisms and Cell Cultures (DSMZ) towards immunogenic wheat proteins with a focus on amylase-trypsin inhibitors (ATIs) and gliadins. The research showed that investigated LAB isolates possess minor differences in their ATIs degradation potential, while gliadin degradation varied strongly. The highest capacity was exhibited by *Lp. plantarum* Lpl5, and Lpl7, as well as *Lac. paracasei* Lpl4 strains.

Dairy products are other consumables that are rich in LAB. The diversity of LAB species present in spontaneously fermented dairy products is significant and affected by the geographical origin and type of the fermented dairy product [12]. Petrova et al. [13] give an overview of LAB consortia in Bulgarian dairy products. *Lactobacillus delbrueckii* ssp. *bulgaricus* in symbiosis with *Streptococcus thermophilus* are usually used as starter cultures in all Bulgarian dairy products, except in koumiss. The accompanying microflora found in these products was highly diverse. The majority belongs to the genera *Lactococcus* and *Leuconostoc* followed by *Entherococcus*, *Pediococcus,* and *Weissela*. Kefir and koumiss also contain yeasts from *Saccharomyces* and *Kazachtania* species. The greatest diversity of microorganisms exists in kefir, followed by yogurt, cheese, koumiss, and katak, and less diversity was observed in kashkaval (a type of yellow cheese).

The antimicrobial properties of some LAB species such as *Bacillus* spp. against Gram-negative bacteria are well known and documented [14]. Usually, *Bacillus* spp. with such properties are isolated from soil, but this species may also be found in various environments, such as plant rhizosphere, human tracts, and foodstuffs [14]. Pajčin et al. [15] demonstrate that antimicrobial lipopeptides produced by *Bacillus velezensis* IP22 isolated from cheese possess antimicrobial properties against the plant pathogen *Xanthomonas euvesicatoria*.

LAB also play an essential role in beverage production and, when certain strains are added, may enhance beverage quality and properties. Zokaityte et al. [16] examined the quality of beverages derived from milk permeate (MP) with (AppMP) or without (MP) the addition of 8% (*w/w*) apple by-products when some LAB strains with antimicrobial properties were added. MP beverages fermented with strains *Liquorilactobacillus uvarum* LUHS245 and *Lacticaseibacillus casei* LUHS210 and AppMP fermented with *Loigolactobacillus coryniformis* LUHS71 showed the highest antimicrobial activity, inhibiting 13 out of 15 tested microbial pathogens. The content of galactooligosaccharide in fermented MP was found to depend significantly on the type of LAB strain, and *Pediococcus acidilactici* LUHS29 was found to be the dominant strain regarding this feature.

LAB from the genus *Carnobacterium* spp. are naturally present in the microflora of seafood, dairy products, and meat [17]. Some, such as *Carnobacterium divergens* and *Carnobacterium maltaromaticum,* may act as food spoilage bacteria when food is improperly stored. Bacteria from the genus *Carnobacterium* are also known as bacteriocins producers with the potential to be used as food antimicrobials. Bergem et al. [18] investigated the antimicrobial activity of 260 *Carnobacterium* spp. isolates from seafood products, including cod, smoked salmon, and shrimp, finding that *C. maltaromaticum* was the most abundant. Other strains belong to *C. divergens*, *C. jeotgali*, *C. inhibens*, *C. funditum*, and *C. viridans*. None were found to be effective against *Candida albicans* and *Pseudomonas fluorescens. C. maltaromaticum* was effective against *Enterococcus faecalis*, *Vibrio parahaemolyticus, Escherichia coli, Brochothrix thermosphacta, Carnobacterium divergens, Lactococcus garvieae, Vagococcus salmoninarum, Listeria monocytogenes,* and *Listeria innocua*, but it was not effective against Gram-negative bacteria and *Staphylococcus epidermidis. C. inhibens* MIP2551 was the only strain effective against *Aeromonas salmonicida*, *Vibrio harveyi*, and *Chromobacterium violaceum.*
